# Anti-Trypanosomal and Antimalarial Properties of Tetralone Derivatives and Structurally Related Benzocycloalkanones

**DOI:** 10.3390/medicina55050206

**Published:** 2019-05-24

**Authors:** Richard M. Beteck, Lesetja J. Legoabe, Michelle Isaacs, Setshaba D. Khanye, Dustin Laming, Heinrich C. Hoppe

**Affiliations:** 1 Centre of Excellence for Pharmaceutical Sciences, North-West University, Potchefstroom 2520, South Africa; 2 Centre for Chemico- and Biomedicinal Research, Rhodes University, Grahamstown 6140, South Africa; m.isaacs@ru.ac.za (M.I.); s.khanye@ru.ac.za (S.D.K.); h.hoppe@ru.ac.za (H.C.H.); 3 Division of Pharmaceutical Chemistry, Faculty of Pharmacy, Rhodes University, Grahamstown 6140, South Africa; 4 Department of Biochemistry and Microbiology, Rhodes University, Grahamstown 6140, South Africa; dustinlaming89@gmail.com

**Keywords:** sleeping sickness, malaria, hit optimization, anti-trypanosomal, anti-malarial, tetralone, benzocycloalkanone

## Abstract

*Background and objectives*: Sleeping sickness and malaria alike are insect-borne protozoan diseases that share overlapping endemic areas in sub-Saharan Africa. The causative agent for malaria has developed resistance against all currently deployed anti-malarial agents. In the case of sleeping sickness, the currently deployed therapeutic options are limited in efficacy and activity spectra, and there are very few drug candidates in the development pipeline. Thus, there is a need to search for new drug molecules with a novel mode of actions. *Materials and Methods*: In the current study, an in vitro screening of a library of tetralone derivatives and related benzocycloalkanones was effected against *T. b. brucei* and *P. falciparum*. *Results*: Several hits with low micromolar activity (0.4–8 µM) against *T. b. brucei* were identified. *Conclusions*: The identified hits have a low molecular weight (<280 Da), a low total polar surface area (<50 Å²), and a defined structure activity relationship, which all make them potential starting points for further hit optimization studies.

## 1. Introduction

Human African trypanosomiasis (HAT), or sleeping sickness, is an insect-borne neglected tropical disease (NTDs) that affects mainly people living in rural areas of sub-Saharan Africa [1]. There are two forms of the disease caused by two subspecies of parasitic protozoans of the genus *Trypanosoma*, species *Trypanosoma brucei* [2]. *T. b. gambiense* is the subspecies causing the West African form of HAT, while *T. b. rhodesiense* is the sub-species responsible for the East African form of HAT [3]. Both forms progress into two stages: Stage 1 and 2 [4]. Stage 1, also referred to as the hemolymphatic stage, is characterized by the presence of parasites in blood and lymphatic circulation [5]. This stage is devoid of any specific signs and symptoms and often progresses unnoticed in cases of *T. b. gambiense* infection [6]. More importantly, stage 1 of a *T. b. rhodesiense* infection is often associated with more serious symptoms, such as severe itching of the skin, irregular fever, and enlargement of the spleen, which can often lead to death [7]. On the other hand, stage 2 (meninogoencephalytic stage) occurs when the parasites, by a yet to be elucidated mechanism, cross the blood–brain barrier and invade the central nervous system. This stage is responsible for a wide range of symptoms including headaches, hallucinations, tremors, sleeping disorders, and convulsions [6]. The progression from stage 1 to 2 occurs within weeks with *T. b. rhodesiense* and months to years with *T. b. gambiense* [8].

Approximately 1500 new cases of HAT were reported in 2017 [9], with *T. b. gambiense* responsible for more than 90% of these cases, while less than 10% could be attributed to *T. b. rhodesiense* infection [10], and it is estimated that 70 million people located in 36 different countries are at risk of being affected by the disease [11]. In addition to mental disabilities, both forms of HAT eventually lead to death if not treated [12].

**Figure 1:**
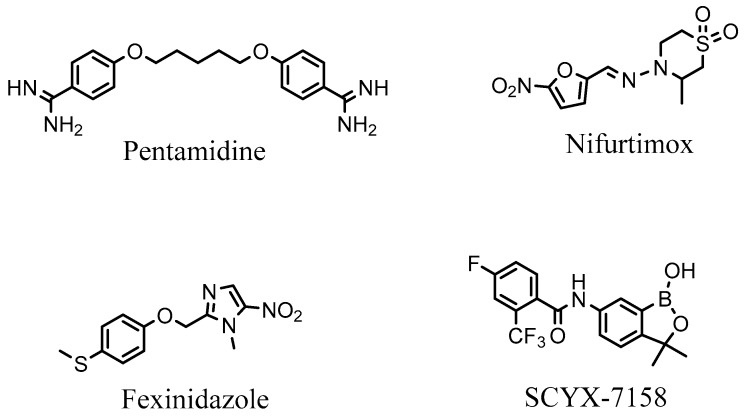
The structures of anti-trypanosomal drugs and candidates under development.

The current treatment regimen for HAT is a rigid one, often made worse by the fact that the activity spectra of currently deployed drugs are very limiting. With no single drug showing activity against neither the forms nor both stages of the disease [13], there is just a single drug available in any case of HAT. For example, in situations of *T. b. gambiense* infection, pentamidine (presented in Figure 1.) is the sole drug available for treating stage 1 of the disease [14], while stage 2 can be treated using only a combination of nifurtimox and eflornithine [15]. Treatment of stages 1 and 2 of *T. b. rhodesiense* infection is possible only with suramin and melarsoprol, respectively [16]. Moreover, these drugs are associated with severe side effects that in some cases kill faster than the disease itself [17].

There is currently a dearth of potential HAT treatments in the development pipeline, with only fexinidazole—a nitroimidazole-based ligand, and SCYX-7158—an oxaborole-based compound (see Figure 1)—presently under clinical development [18]. It is therefore paramount to search for alternative treatment options for HAT.

In addition to *T. b. rhodesiense* and *T. b. gambiense*, *T. b. brucei* is the third subspecies of *Trypanosoma brucei*, and it is one of the parasites responsible for Nagana—a related form of HAT in livestock [19]. Nagana in cattle leads to loss in meat and milk production, which is associated with great economic consequences [20]. Like HAT, Nagana is currently managed using just a few drugs (diminazene and isometamidium chloride) against which resistant parasites have emerged [21]. In addition to causing Nagana, *T. b. brucei* serves as a model for drug discovery against HAT [22].

Malaria is another insect-borne disease of high burden to sub-Saharan Africa. In 2017, it reportedly led to the death of 435,000 people worldwide, and 93% of these deaths occurred in sub-Saharan Africa, where a child under the age of five is lost to malaria every two minutes [23]. Although the number of deaths attributed to malaria has decreased over the years since 2000 [24], it is still a great global health threat with some regions continuously witnessing increasing drug resistant strains [25]. This situation jeopardizes the efficacy of currently deployed anti-malarials [26]. Malaria treatment and prophylaxis rely on the use of chemotherapeutic agents [27]. There are just five classes of such agents so far in the long history of malarial chemotherapy, including aminoquinolines, aminoalcohols, anti-folates, hydroxynapthoquinone, and endoperoxides [28]. It is important to note that *P. falciparum*, the parasite responsible for almost 90% of malaria cases in sub-Saharan Africa [29], has developed resistance against all these drug classes even when they are deployed in combination therapy. This suggests that *P. falciparum* has an inbuilt capacity to develop resistance against any drug(s) [30] and places emphasis on the continued search for new compounds with antimalarial properties.

Cross-screening, the practice of screening known hits against other targets or diseases, has been hailed as a cost-effective approach for hit and lead generation [31]. A good example of this practice relates to the malarial box, which is a collection containing 400 compounds initially synthesized and investigated for antimalarial activity [32]. The malarial box has been extensively cross-screened against several targets, culminating in new hits in different therapeutic areas including tuberculosis and sleeping sickness [33]. It is also worth mentioning the work by Monti et al. wherein compounds exhibiting potent activity against *T. b. brucei* were identified by screening compounds previously synthesized and investigated as potential hits against Alzheimer’s disease [34].

In this work, we report the anti-trypanosomal, antimalarial, and cytotoxicity properties of a series of tetralone derivatives and related benzocycloalkanones. The structure activity relationships (SAR) were also analysed. The compounds in this study were previously synthesized and investigated as inhibitors of monoamines-oxidase (MAO), a viable target for the management of Alzheimer’s disease [35,36].

## 2. Materials and Methods

### 2.1. In Vitro Anti-Trypanosomal Assay

The activity of the compounds against 427 *Trypanosoma brucei brucei* trypomastigotes was determined as previously described [37]. Briefly, parasites were incubated in 96-well plates with 20 µM or three-fold serial dilutions (100 µM starting concentration) of test compounds in Iscove’s modified Dulbecco’s medium (IMDM) (Lonza, Basel, Switzerland) supplemented with 10% fetal calf serum, HMI-9 supplement, hypoxanthine, and penicillin/streptomycin at 37 °C in a 5% CO_2_ incubator. After 48 h, 20 µL of resazurin reagent (0.135 mg/mL resazurin in phosphate-buffered saline) was added to each well and the fluorescence (Ex_560_/Em_590_) read after 2–4 h in a Spectramax M3 plate reader (Molecular Devices, San Jose, CA, USA). The fluorescence readings were used to calculate % parasite viability relative to the readings obtained from the wells containing untreated control parasite cultures. The IC_50_ values were determined by plotting % viability vs. log(compound) and performing a non-linear regression using a GraphPad Prism (v. 5.02) (San Diego, CA, USA).

### 2.2. In Vitro Cytotoxicity Assay

As previously described [17], HeLa cells (Cellonex, Johannesburg, South Africa) were seeded in 96-well plates at a density of 2 × 10^4^ cells/well in Dulbecco’s modified Eagle’s medium (DMEM) (Lonza, Basel, Switzerland) supplemented with 10% fetal calf serum and antibiotics (penicillin/streptomycin/amphotericin B) and incubated overnight at 37 °C in a 5% CO_2_ incubator. Compounds were added to the cells at a final concentration of 20 µM, and incubation continued for 24 h. The cell viability in the wells was assessed by adding 20 µL of resazurin reagent (0.135 mg/mL resazurin in phosphate-buffered saline) and reading the fluorescence (Ex560/Em590) after an additional 2–4 h incubation. The fluorescence readings were converted to % cell viability relative to the average readings obtained from the untreated control wells.

### 2.3. In Vitro Antiplasmodial Assay

An antimalarial evaluation was performed against the chloroquine sensitive strain of *P. falciparum* (3D7) using the parasite lactate dehydrogenase (pLDH) assay as previously reported [38]. Parasites were seeded at 2% parasitemia and 1% hematocrit in 96 well plates and were incubated with 20 µM of the test compounds for 48 h in culture medium consisting of Rosell Park Memorial Institute (RPMI 1640; Lonza, Basel, Switzerland) containing 25 mM 4-(2-hydroxyethyl)-1-piperazineethanesulfonic acid (HEPES), supplemented with 0.5% (*w*/*v*) Albumax II, 22 mM glucose, 0.65 mM hypoxanthine, 0.05mg/mL gentamicin, and 1% (*v*/*v*) human erythrocytes, at 37 °C under an atmosphere of 5% CO_2_, 5% O_2_, and 90% N_2_. The parasite lactate dehydrogenase activity in the wells was determined using the colorimetric assay method of Makler et al. [39]. The absorbance readings were converted to % parasite viability relative to the wells containing untreated parasites.

## 3. Results

**Figure 2:**
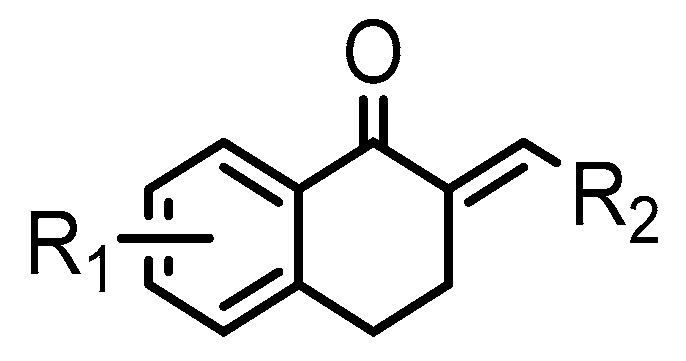
General structure for tetralones deployed in this study.

**Figure 3:**
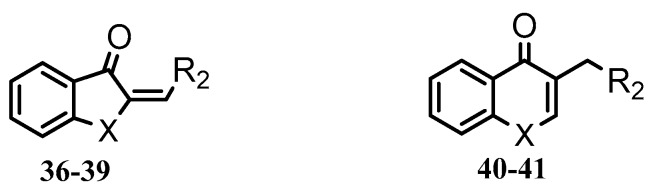
General structure for benzocycloalkanones deployed in this study.

Thirty-five tetralone derivatives (see Figure 2) and six related benzocycloalkanones (see Figure 3) were evaluated in vitro against *T. b. brucei* for antitrypanosomal activity through the resazurin assay protocol. The compounds were also evaluated against the chloroquine-sensitive strain (3D7) of *P. falciparum* for antimalarial activity using pLDH assay and against the HeLa cell line for cytotoxicity evaluation using the resazurin assay protocol. Initially, a single point assay using 20 µM solution of the target compound against the biological targets of interest was carried out, and the results were reported as percent (%) viability (see Table 1 and Table 2 below), which is the percentage of cells remaining viable after incubation with each compound relative to the untreated cells. Any compound exhibiting a percent viability below 25% against *T. b. brucei* and/or 3D7 was earmarked for further dose-response analyses, provided it showed a HeLa cell viability greater than 50%.

With regards to antitrypanosomal activity, twenty six out of thirty-five (26/35) tetralone derivatives exhibited a less than 25% *T. b. brucei* cell viability, while three out of six benzocycloalkanones reduced the percentage parasite viability to <25%. These compounds were advanced to determine the corresponding IC_50_ values (Table 3). In total, twelve compounds were subjected to IC_50_ determination, and they exhibited potent anti-trypanosomal activity in the range of 0.4 to 6.7 µM. In addition to their low micromolar activity, hit compounds had a low molecular weight (<300 Da) and a low total polar surface area (<50 Å^2^), which make them suitable starting points for hit-to-lead optimization studies—a process that often leads to an increased molecular weight and a total polar surface area (*tPSA)*. Identified hits, however, had high ClogP values (>3), which can be resolved through structural incrementation with polar moieties. 

The tetralone derivatives and related benzocycloalkanones were also evaluated against the chloroquine-sensitive strain of *Plasmodium falciparum* (3D7) for antimalarial activity. Most of the compounds investigated showed poor antimalarial activity, exhibiting a 3D7 viability of greater than 25% at 20 µM (Figures S1 and S2). Only seven compounds in this series exhibited a 3D7 viability of less than 25%.

This series generally showed a low cytotoxicity effect as measured against the Hela cell line, with only ten out of forty-one compounds exhibiting a Hela cell viability below 25% at 20 µM. Most of the compounds demonstrated a Hela cell viability greater than 50%, which indicates they pose little toxicity risk and are far from acting as frequent hitters or pan-assay interference (PAINS) compounds.

## 4. Discussions

As illustrated by the data presented in Table 1 and Table 3, it can be observed that the structural variation around the benzoid ring (R_1_) and/or the functionalization of the cyclohexanone ring of the tetralone scaffold influenced the anti-trypanosomal activity. With regards to the benzoid ring (R_1_), the presence of -NH_2_ attached to the benzoid ring of tetralone generally led to compounds with reduced anti-trypanosomal activity. For example, compound 1, devoid of an -NH_2_ moiety, exhibited a low parasite % viability of 0.06 and a low IC_50_ value of 2.3 µM, while its congener compound 2, which incorporated -NH_2_ at position 7 of the benzoid ring, had poor anti-trypanosomal activity with 95% of the parasites still being viable at 20 µM. This observation follows suit with compound 7, which had a low parasite % viability of 0.66 and a very potent IC_50_ value of 0.4 µM compared to its -NH_2_ bearing analogue compound 11, which exhibited a high parasite viability of 95% at 20 µM. Other benzoid ring substituents (R_1_), such as -OH, -OCH_3_, and -H, favored anti-trypanosomal activity, with the -OH moiety giving the best activity. This is evident when comparing compound 7 (IC_50_ = 0.4 µM) against compound 3, which showed a moderate parasite viability of 43% at 20 µM, and compound 12 demonstrating submicromolar activity (IC_50_ = 0.6 µM) against compound 5, which had no inhibitory effect on the parasites at 20 µM.

Concurrently with the benzoid substituents, substituents attached to the cyclohexanone ring (R_2_) also influenced anti-trypanosomal activity. The structure activity relationship (SAR) analyses of this series suggested that the functionalization of the cyclohexanone ring of tetralone generally promoted anti-trypanosomal activity. For example, compounds 10, 26, 27, and 31, although they all bore structurally diverse moieties tethered through the cyclohexanone ring, had comparable anti-trypanosomal activity. At 20 µM, they all exhibited a parasite viability below 1%. In general, the data appears to suggest that six membered heteroaromatic rings including cyclohexane and cyclopentane exhibited good anti-trypanosomal activity, while five membered heteroaromatic ring systems including furan, thiophene, and pyrrole exhibited moderate anti-trypanosomal activity.

In addition to tetralone, other structurally related ring systems including indanone (36), benzosuberone (37), chromanone (38) thiochromanone (39), chromone (40), and thiochromone (41) were also investigated for antitrypanosomal activity, and their inhibitory effects at 20 µM are summarized in Table 2. Comparing the structure and activity of compound 24, a tetralone derivative that exhibited a parasite viability of 26% at 20 µM against compounds 36, 40, and 41—all of which exhibited a parasite viability >90% at 20 µM—it posits that the tetralone scaffold enhanced antitrypanosomal activity compared to indanone, chromone, and thiochrmone. Furthermore, compounds 37, 38, and 39 exhibited a lower parasite viability (<1%) than compound 24 at 20 µM, which suggests that the benzosuberone, chromanone, and thiochromanone scaffolds possessed anti-trypanosomal activity and are worth further exploration. Compound 38, a derivative of chromanone, possessed potent activity against *T. b. brucei* with an IC_50_ value of 0.4 µM (see Table 3).

With respect to the observed antimalarial activity, comparing the structure and activity of compounds 8, 12, and 14 for example—all of which exhibited a 3D7 viability below 25%—it suggests that the concurrent substitution of the benzoid ring (R_1_) with an -OH group and the substitution of the cyclohexanone ring (R_2_) with a phenyl unit was required for antimalarial activity.

## 5. Conclusions

A series of tetralone derivatives and related benzocycloalkanones previously investigated as inhibitors of monoamine oxidase were cross-screened against *T. b. brucei* and *P. falciparum* in search of new anti-trypanosomal and antimalarial agents, respectively. At 20 µM, only a few compounds showed promising antimalarial activity, while about 70% of the compounds tested showed promising anti-trypanosomal activity—exhibiting a less than 25% *T. b. brucei* viability. In other words, these compounds inhibited more than 75% of *T. b. brucei* growth at 20 µM. The low IC_50_ values demonstrated by some compounds in this series, coupled with their low molecular weight and total polar surface area (*tPSA*), make them ideal hits for further optimization.

## Figures and Tables

**Table 1:** The structures of R_1_, R_2_, and % viability of *T.b. brucei*, *P. falciparum* (3D7), and Hela cells at 20 µM of tested tetralones.

Compound	R_1_	R_2_	% Viability at 20 µM (SD)		
			*T.b. brucei*	3D7	HeLa Cell
1	-	3-CN-phenyl	0.06(0.3)	113 (3)	87 (3)
2	7-NH_2_	3-CN-phenyl	95 (3)	110 (1)	77 (2)
3	-	phenyl	43 (3)	103 (2)	116 (5)
4	-	4-OCH_3_-phenyl	0.5 (0.5)	100 (9)	76 (9)
5	-	4-F-phenyl	101 (1)	107 (5)	100 (2)
6	-	3-F-phenyl	0.5 (0.2)	131 (9)	121 (16)
7	6-OH	phenyl	0.6 (0.2)	89 (4)	54 (1)
8	5-OH	phenyl	0.5 (0.1)	21 (12)	35 (1)
9	7-OH	phenyl	−4 (0.3)	101 (4)	9 (0.1)
10	7-OCH_3_	phenyl	0.1 (0.1)	113 (1)	65 (3)
11	6-NH_2_	phenyl	97 (0.3)	111 (1)	86 (9)
12	7-OH	4-F-phenyl	−0.6 (0.1)	11 (6)	29 (0.1)
13	7-OH	3-F-phenyl	−0.9 (0.1)	13 (1)	21 (1)
14	7-OH	4-Cl-phenyl	0.1 (0.1)	13 (14)	27 (2)
15	7-OH	3-Cl-phenyl	0.6 (0.01)	−3 (6)	18 (0.4)
16	7-OH	4-Br-phenyl	0.1 (0.1)	43 (8)	62 (2)
17	-	3-Cl-phenyl	0.2 (0.3)	100 (9)	41 (13)
18	7-OH	3-Br-phenyl	1.2 (0.31)	99 (8)	26 (3)
19	7-OH	4-OH-phenyl	1 (0.2)	104 (12)	44 (4)
20	7-OH	4-CH_3_-phenyl	−0.4 (0.1)	116 (16)	69 (10)
21	7-OH	4-N(CH_3_)_2_-phenyl	25 (2)	142 (9)	51 (4)
22	7-OH	3,4-diCl-phenyl	0.9 (0.2)	81 (1)	5 (0.9)
23	7-OH	4-OCH_3_-phenyl	0.2 (0.1)	88 (10)	25 (2)
24	-	4-Cl-phenyl	26 (8)	100 (8)	100 (3)
25	-	3-pyridyl	−0.7 (0.4)	29 (12)	24 (0.4)
26	7-OCH_3_	cyclohexyl	0.8 (0.5)	119 (13)	92 (16)
27	7-OCH_3_	cyclopentyl	0.6 (0.02)	81 (3)	85 (1.6)
28	7-OCH_3_	furanyl	31 (5)	100 (3)	97 (9)
29	7-OCH_3_	pyrrolyl	103 (1)	115 (5)	97 (5)
30	7-OCH_3_	2-thiophenyl	32 (9)	107 (6)	106 (2)
31	7-OCH_3_	3-pyridyl	0.2 (0.03)	85 (12)	17 (4)
32	7-OCH_3_	4-pyridyl	−0.1 (0.7)	36 (12)	23 (2)
33	7-OCH_3_	2-pyridyl	−0.4 (0.2)	7 (8)	20 (0.6)
34	7-OCH_3_	2-Cl-3-pyridyl	98 (2)	113 (6)	47 (1.7)
35	7-OCH_3_	3-thiophenyl	22 (2)	86 (10)	122 (1)

**Table 2:** The structures of X, R_2_, and % viability of *T.b. brucei*, *P. falciparum* (3D7), and Hela cells at 20 µM of tested benzocycloalkanones.

Compound	X	R_2_	% Viability at 20 µM (SD)		
			*T. b. brucei*	3D7	HeLa Cell
24	(CH_2_)_2_	4-Cl-phenyl	26 (8)	100 (8)	100 (3)
36	CH_2_	4-Cl-phenyl	99 (0.1)	93 (3)	74 (10)
37	(CH_2_)_3_	4-Cl-phenyl	0.3 (0.3)	113 (0.6)	53 (10)
38	OCH_2_	4-Cl-phenyl	0.4 (0.2)	98 (1)	75 (5)
39	SCH_2_	4-Cl-phenyl	0.3 (0.3)	11 (0.9)	8 (2)
40	O	4-Cl-phenyl	94 (7)	90 (6)	103 (3)
41	S	4-Cl-phenyl	103 (0.3)	107 (5)	109 (7)

**Table 3:** The IC_50_ values of the selected compounds and pentamidine against *T.b. brucei*.

Compound	Structure	IC_50_ (µM)	MW ^a^	ClogP ^b^	*tPSA* ^c^
		*T. b. brucei*			
1	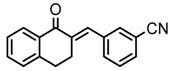	2.3	259	3.5	40
4	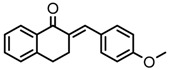	6.7	264	4	26
6	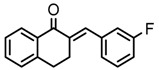	3.1	252	4	17
7	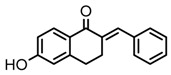	0.46	250	4	37
10	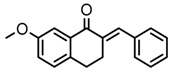	1.04	264	4	26
12	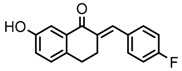	0.68	268	4	37
16	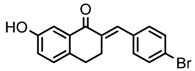	1.24	329	4.8	37
20	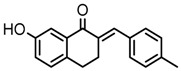	5.3	264	4.5	37
26	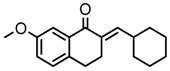	nd	270	5.6	26
27	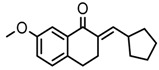	5.4	256	5	26
35	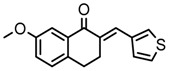	2.6	270	4	26
38	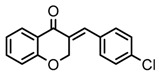	0.4	270	4.4	26
PE	-	0.0042	-	-	-

PE is pentamidine, MW is the molecular weight, ClogP is the calculated lipophilicity, *tPSA* is the total polar surface area, and nd is not determined. a, b, and c were all generated using ACD Chemsketch Freeware Version 12.

## Supplementary Materials

The following are available online at https://www.mdpi.com/1010-660X/55/5/206/s1: Figure S1. shows the dose response curve for the selected compounds and pentamidine against *T. b. brucei.*, and Figure S2. Shows the dose response curve for the selected compounds and pentamidine against *T. b. brucei.*

## References

[1] Brun R., Schumacher R., Schmid C., Kunz C., Burri C. (2001). The phenomenon of treatment failures in human African trypanosomiasis. Trop. Med. Int. Health.

[2] Barrett M., Burchmore R., Stich A., Lazzari J., Frasch A., Cazzulo J., Krishna S. (2003). The trypanosomiases. Lancet.

[3] Beteck R.M., Legoabe L.J., Isaacs M., Hoppe H.C. (2018). In vitro Anti-trypanosomal activities of indanone-based chalcones. Drug Res..

[4] Kaiser M., Bray M., Cal M., Trunz B., Torreele E., Brun R. (2011). Antitrypanosomal activity of fexinidazole, a new oral nitroimidazole drug candidate for treatment of sleeping sickness. Antimicrob. Agents Chemother..

[5] MacLean L., Reiber H., Kennedy P., Sternberg J. (2012). Stage progression and neurological symptoms in trypanosoma brucei rhodesience sleeping sickness: Role of the CNS inflammatory response. PLoS Negl. Trop. Dis..

[6] Kennedy P.G. (2004). Human African trypanosomiasis of the CNS: Current issues and challenges. J. Clin. Investig..

[7] Stich A., Abel P., Krishna S. (2002). Human African trypanosomiasis. BMJ.

[8] Checchi F., Filipe J., Haydon D., Chandramohan D., Chappuis F. (2008). Estimates of the duration of the early and late stage of gambiense sleeping sickness. Infect. Dis..

[9] Global Health Observatory Data. http://www.who.int/gho/neglected_diseases/human_african_trypanosomiasis/en/.

[10] Kuepfer I., Hhary E., Allan M., Edielu A., Burri C., Blum J. (2011). Clinical presentation of T.b. rhodesiense sleeping sickness in second stage patients from Tanzania and Uganda. PLoS. Negl. Trop. Dis..

[11] Simarro P., Cecchi G., Franco J., Paone M., Diarra A., Ruiz-Postigo J., Fe’vre E., Mattioli R., Jannin G. (2012). Estimating and mapping the population at risk of sleeping sickness. PLoS. Negl. Trop. Dis..

[12] Franco J.R., Simarro P.P., Diarra A., Jannin J.G. (2014). Epidemiology of human African trypanosomiasis. Clin. Epidemiol..

[13] Babokhov P., Sanyaolu A., Oyibo W., Fagbenro-Beyioku A., Iriemenam N. (2013). A current analysis of chemotherapy strategies for the treatment of human African trypanosomiasis. Pathog. Glob. Health.

[14] Song J., Baker N., Rothert M., Henke B., Jeacock L., Horn D., Beitz E. (2016). Pentamidine is not a permeant but a nanomolar inhibitor of the Trypanosoma brucei aquaglyceroporin-2. PLoS Pathog..

[15] Alirol E., Schrumpf D., Amici Heradi J., Riedel A., de Patoul C., Quere M., Chappuis F. (2013). Nifurtimox-eflornithine combination therapy for second-stage gambiense human African trypanosomiasis: Médecins San Frontières experience in the Democratic Republic of Congo. Clin. Infect. Dis..

[16] Steverding D. (2010). The development of drugs for treatment of sleeping sickness: A historical review. Parasit. Vectors.

[17] Beteck R.M., Isaacs M., Hoppe H.C., Khanye S.D. (2018). Synthesis, in vitro cytotoxicity and trypanocidal evaluation of novel 1,3,6-substituted non-fluoroquinolones. S. Afr. J. Chem..

[18] Berninger M., Schmidt I., Ponte-Sucre A., Holzgrabe U. (2017). Novel lead compounds in pre-clinical development against African sleeping sickness. Med. Chem. Commun..

[19] Troeberg L., Chen X., Flaherty T., Morty R., Cheng M., Hua H., Springer C., McKerrow J., Kenyon G., Lonsdale-Eccles J. (2000). Chalcone, acyl hydrazide, and related amides kill cultured Trypanosoma brucei brucei. Mol. Med..

[20] Connor R.J. (1994). The impact of nagana. Onderstepoort. J. Vet. Res..

[21] Giordani F., Morrison L., Rowan T., de Koning H., Barrett M. (2016). The animal trypanosomiases and their chemotherapy: A review. Parasitology.

[22] Veale C.G., Hoppe H.C. (2018). Screening of the Pathogen Box reveals new starting points for anti-trypanosomal drug discovery. Med. Chem. Commun..

[23] WHO (2018). World Malaria Report 2018.

[24] Meyrowitsch D., Pedersen E., Alifrangis M., Scheike M., Malecela M., Magesa S., Derua Y., Rwegoshora R., Michael E., Simonsen P. (2011). Is the current decline in malaria burden in sub-Saharan Africa due to a decrease in vector population?. Mal. J..

[25] Roberts L. (2017). Drug-resistant malaria advances in Mekong. Science.

[26] Dondorp A.M., Nosten F., Yi P., Das D., Phyo A.P., Tarning J., Lwin K.M., Ariey F., Hanpithakpong W., Lee S.J. (2009). Artemisinin resistance in Plasmodium falciparum malaria. N. Engl. J. Med..

[27] Schwartz E. (2012). Prophylaxis of Malaria. Mediterr. J. Hematol. Infect. Dis..

[28] Na-Bangchang K., Karbwang J. (2009). Current status of malaria chemotherapy and the role of pharmacology in antimalarial drug research and development. Fundam. Clin. Pharmacol..

[29] Enato E.F., Okhamafe A.O. (2005). Plasmodium falciparum malaria and antimalarial interventions in sub-Saharan Africa: Challenges and opportunities. Afr. J. Biotechnol..

[30] Verlinden B.K., Louw A., Birkholtz L.M. (2016). Resisting resistance: Is there a solution for malaria?. Expert Opin. Drug Discov..

[31] Devine W., Woodring J.L., Swaminathan U., Amata E., Patel G., Erath J., Roncal N.E., Lee P.J., Leed S.E., Rodriguez A. (2015). Protozoan parasite growth inhibitors discovered by cross-screening yield potent scaffolds for lead discovery. J. Med. Chem..

[32] Spangenberg T., Burrows J.N., Kowalczyk P., McDonald S., Wells T.N., Willis P. (2013). The open access malaria box: A drug discovery catalyst for neglected diseases. PLoS ONE.

[33] Van Voorhis W.C., Adams J.H., Adelfio R., Ahyong V., Akabas M., Alano P., Alday A., Resto Y., Alsibaee A., Alzualde A. (2016). Open source drug discovery with the malaria box compound collection for neglected diseases and beyond. PLoS Pathog..

[34] Monti L., Wang S., Oukoloff K., Smith A., Brunden K., Caffrey C., Ballatore C. (2018). Brain-penetrant, triazolopyrimidine and phenylpyrimidine microtubule-stabilizers as potential leads to treat Human African Trypanosomiasis. ChemMedChem.

[35] Amakali K.T., Legoabe L.J., Petzer A., Petzer J.P. (2018). Synthesis and in vitro evaluation of 2-heteroarylidene-1-tetralone derivatives as monoamine oxidase inhibitors. Drug Res. (Stuttg.).

[36] Amakali K.T., Legoabe L.J., Petzer A., Petzer J.P. (2018). Synthesis and valuation of 2-benzylidene-1-tetralone derivatives for monoamine oxidase inhibitory activity. Cent. Nerv. Syst. Agents Med. Chem..

[37] Gumbo M., Beteck R.M., Mandizvo T., Seldon R., Warner D.F., Hoppe H.C., Isaacs M., Laming D., Tam C.C., Cheng L.W. (2018). Cinnamoyl-Oxaborole Amides: Synthesis and Their in Vitro Biological Activity. Molecules.

[38] Darrell O.T., Hulushe S.T., Mtshare T.E., Beteck R.M., Isaacs M., Laming D., Hoppe H.C., Krause R.W.M., Khanye S.D. (2018). Synthesis, antiplasmodial and antitrypanosomal evaluation of a series of novel 2-oxoquinoline-based thiosemicarbazone derivatives. S. Afr. J. Chem..

[39] Makler M.T., Ries J.M., Williams J.A., Bancroft J.E., Piper R.C., Gibbins B.L., Hinrichs D.J. (1993). Parasite lactate dehydrogenase as an assay for *Plasmodium falciparum* drug sensitivity. Am. J. Trop. Med. Hyg..

