# Supplementary material for: Anti-Trypanosomal and Antimalarial Properties of Tetralone Derivatives and Structurally Related Benzocycloalkanones
Source: Medicina (Kaunas). 2019 May 24;55(5):206. doi: 10.3390/medicina55050206 (PMC6572618; doi:10.3390/medicina55050206)
Supplement: Supplementary file 1 [file medicina-55-00206-s001.pdf]

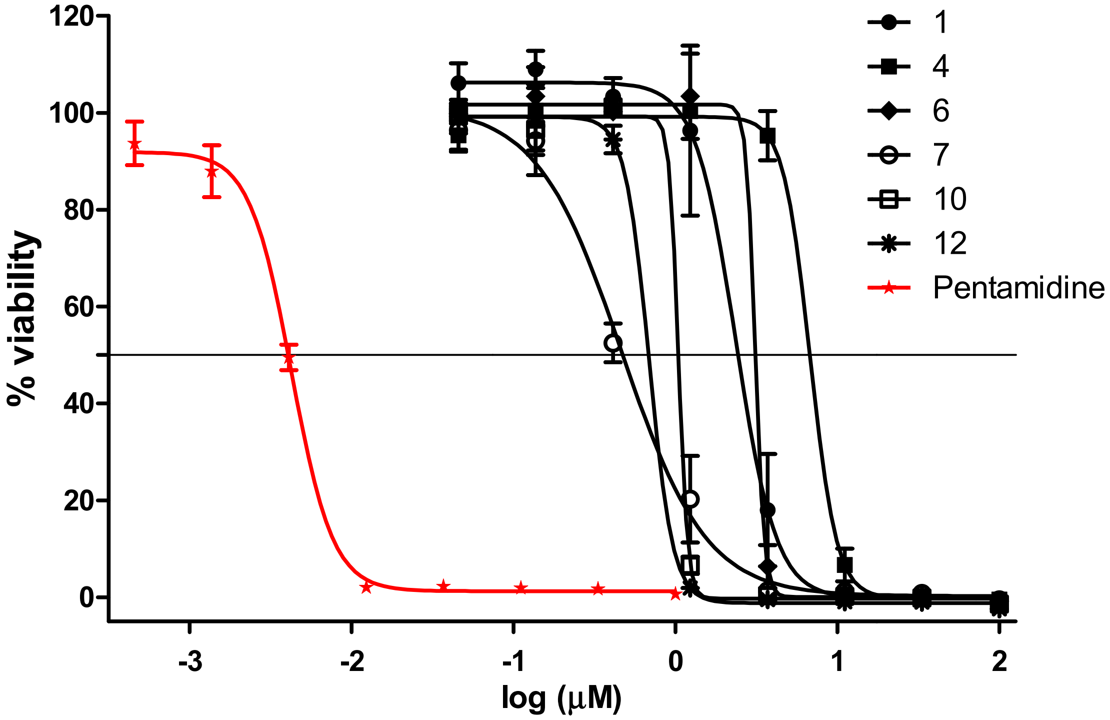

Figure S1. shows dose response curve for selected compounds and pentamidine against *T. b. brucei*.

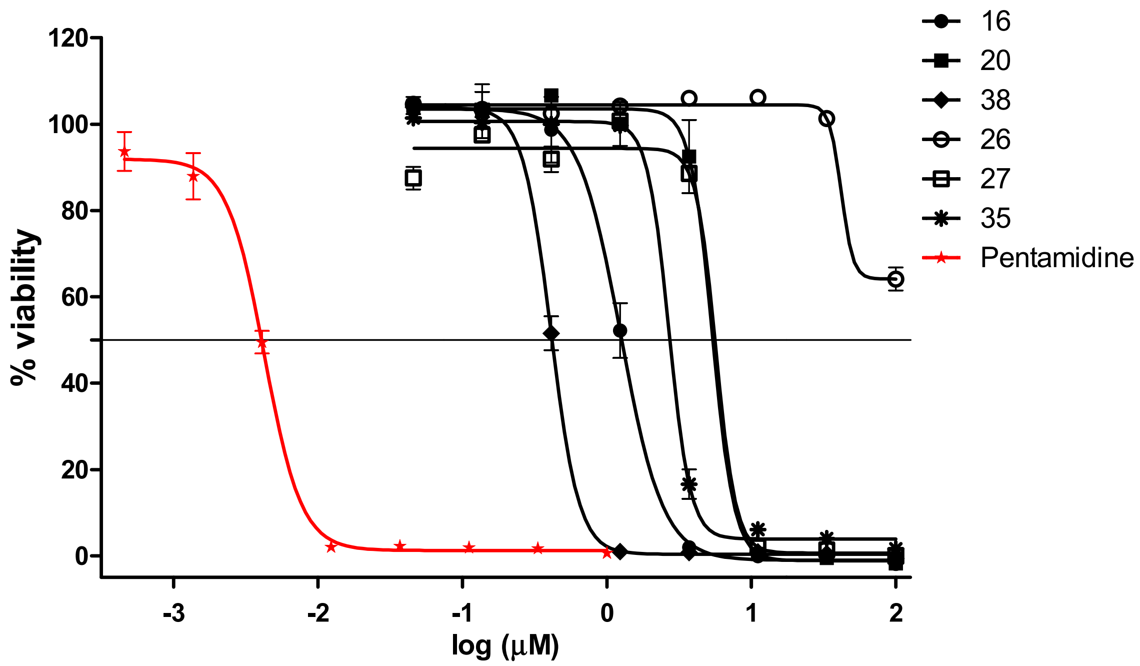

Figure S2. shows dose response curve for selected compounds and pentamidine against *T. b. brucei*.
